# Impact of Early Admission Surveys on Physicians’ Communication Skills on Hospital Consumer Assessment of Healthcare Providers and Systems (HCAHPS) Scores: A Quality Improvement Initiative

**DOI:** 10.7759/cureus.68616

**Published:** 2024-09-04

**Authors:** David Bryant, Mahesh Bhattarai, Austin Clark, Madison Mclnnis, Farnaz Solatikia, Brittany Stith, Lee Justin, Hussam M Ammar

**Affiliations:** 1 Internal Medicine, Tallahassee Memorial Hospital/Florida State University, Tallahassee, USA; 2 Internal Medicine, Florida State University College of Medicine, Tallahassee, USA; 3 Biostatistics and Epidemiology, Florida State University College of Medicine, Tallahassee, USA; 4 Research, Tallahassee Memorial Hospital, Tallahassee, USA; 5 Research, Tallahassee Memorial Hospital/Florida State University, Tallahassee, USA

**Keywords:** explain things in a language that patients understand, listen to the patient, treat with respect, doctrors' patients communication skills, double surveying, hcahps communication score

## Abstract

Introduction

Effective communication between doctors and patients is one of the 19 core questions on the Hospital Consumer Assessment of Healthcare Providers and Systems (HCAHPS) survey. Positive patient experiences, as reflected in HCAHPS scores, are associated with higher quality of care, reduced mortality, and fewer readmissions. These scores also influence a hospital’s financial bonuses or penalties from the Centers for Medicare & Medicaid Services.

Methods

This quality improvement (QI) initiative evaluated how early surveys of patients’ feedback on doctors’ communication skills during admission impacted discharge communication scores. The approach involved promptly sharing patients’ feedback with physicians and focusing on improving communication skills for those with less-than-perfect scores.

Results

This QI initiative involved 41 patients. By surveying patients early during admission and addressing communication issues for those with less-than-perfect scores, the percentage of patients achieving a perfect score on all survey questions increased from 18 (43.9%) at admission to 34 (82.9%) at discharge. The largest improvement was seen in perfect scores for doctors’ explanations of patients’ conditions, which rose from 59% at admission to 90% at discharge. Conversely, the increase in perfect scores for doctors’ respectful treatment was smaller, rising from 88% at admission to 98% at discharge.

Conclusion

Surveying patients about their doctors’ communication skills early in admission to identify those with less-than-perfect scores and improving communication for this group resulted in a 39% increase in satisfaction with doctors’ communication.

## Introduction

“Listen to the patient; he is telling you the diagnosis.” This quote was attributed to William Osler, who realized the central role of physician-patient communication in patient care almost a century before the Hospital Consumer Assessment of Healthcare Providers and Systems (HCAHPS) was developed [1,2]. Physician-patient communication is one of the cores of the survey. HCAHPS has been implemented nationally by the Centers for Medicare & Medicaid Services (CMS) since 2006, and results were first reported to the public in 2008 [2]. The Patient Protection and Affordable Care Act of 2010 mandated that HCAHPS scores would be used to determine part of the value-based purchasing for hospitals [3]. Thus, in October 2012, the CMS officially started to pay hospitals for the quality of care they provided versus just the quantity of care they offered [2,3].

The HCAHPS survey asks discharged patients 29 questions about their recent hospital stay. The survey contains 19 core questions about critical aspects of patients’ hospital experiences (communication with nurses and doctors, the responsiveness of hospital staff, the cleanliness and quietness of the hospital environment, communication about medicines, discharge information, overall rating of the hospital, and would they recommend the hospital) [2,4]. CMS publishes participating hospitals’ HCAHPS results on the Care Compare website four times yearly. Consumers can compare different hospital surveys. The survey is scored as percentages of top-box (i.e., most positive) responses (i.e., “top-box scores,” “Always” for five HCAHPS domains, “Yes” for discharge information, “9” or “10” for hospital rating, and “Definitely” for recommend the hospital). Multiple studies have positively linked patient experience, as measured by HCAHPS, to a higher quality of care, lower mortality, and fewer readmissions. [2,4,5]. The positive outcomes of high HCAPHS scores were not consistently documented in all research papers. In a nationally representative sample, one study found that higher patient satisfaction was associated with lower emergency department utilization, higher inpatient utilization, more significant total healthcare expenditures, and higher expenditures on prescription drugs. The most satisfied patients also had a statistically significant higher mortality risk than the least satisfied patients [6,7]. Another study conducted by Dartmouth Hitchcock and the University of Michigan suggests that satisfied patients are more likely to struggle with opioid addiction [6]. Physicians’ communication skills significantly impact patient satisfaction and positively correlate with improved patient satisfaction scores. Several interventions have been designed to improve doctors’ communications, such as communication skills training programs for healthcare providers, providing physicians face cards for patients, and providing daily feedback to healthcare providers directly or through emails [8].

Tallahassee Memorial Hospital (TMH) outlined a strategic plan to improve the domains of the HCAHPS survey. The internal medicine residency program (IMRP) at TMH performed a quality improvement (QI) project implementing an innovative intervention: conducting a survey early during admission that mirrors HCAHPHS doctors’ communication domain to identify patients who are not satisfied with healthcare providers’ communication and to focus on this group and then test the intervention by administering a second survey at discharge.

## Materials and methods

We conducted a QI project at TMH, a 772-bed acute care hospital that houses three residencies: general surgery, family medicine, and internal medicine. The IMRP at TMH is ACGME-accredited and sponsored by Florida State University College of Medicine. The IMRP has two inpatient teams; each has a resident, two interns, and an attending. One of the study’s authors (DB) hypothesized that identifying patients who are not satisfied with doctors’ communication early on during the admission by administering a mock survey with three questions doctors’ communication survey in HCAHPS on day one or two will give the physicians a window of opportunity to target this vulnerable group with better communication and ultimately will improve the HCAPHS communication score. Three authors (AC, MB, and DB) delivered two one-hour presentations to enhance IMRP residents’ and faculty’s communication skills based on HCAHPS doctor’s communications survey questions. Table 1 illustrates the techniques discussed with residents and faculty. Educational cards with these instructions were printed and distributed to the residents in the inpatient service. The Institutional Board Review at TMH approved the QI project. The doctor’s communication domain in HCAHPS comprises three questions: (1) How often did the doctors treat you with respect during this hospital stay? (2) During this hospital stay, how often did the doctors listen to you? and (3) During this hospital stay, how often did the doctors explain things in a language you can understand? The survey uses frequency-based questions with possible answers fixed on a 4-point scale (4 = Always, 3 = Usually, 2 = Sometimes, and 1 = Never). The result will be scored positive only if it has an “Always” answer to the three questions [2,3].

**Table 1:** Instructions and techniques to enhance doctors’ communication skills

1. Knock before entering the room.
2. Greet the patient by name and acknowledge others in the room.
3. Introduce yourself and your role and update the whiteboard.
4. Ensure the patient is comfortable and maximizes privacy.
5. Set a shared agenda with the patient.
6. Ask permission before conducting a physical exam.
7. Thank the patient at the end of the encounter.
8. Sit or kneel, and make eye contact, and smile when appropriate.
9. Ask patients to record questions so they don’t forget them.
10. Summarize key points of discussion.
11. Recognize nonverbal cues, body language, and emotions.
12. Show empathy: Clearly explain the plan of care to the patient.
13. Avoid medical jargon.
14. Explain physical exam findings as you perform the exam.
15. Use visual aids, analogies, and diagrams.
16. Explain the testing/procedures before ordering.
17. Describe the plan of care at daily rounds.
18. Ask “What questions do you have?”

The survey was conducted between January 1 and January 31, 2024, on patients admitted to the teaching service at TMH age ≥18; the exclusion criteria included pregnancy, inability to complete the survey because of confusion, altered mental status, incarcerated status, language barriers, and patients admitted under observation status with expected admission less than 48 hours. Fifty patients were initially considered for the survey; nine were excluded because of altered mental status, language barrier, and decline to complete the survey. AC and MB conducted the mock survey 24-48 hours after admission and on discharge day. The project’s authors did not participate in the actual care of the patients. On survey day, the primary team was notified by secure TMH messenger that one of their patients did not answer “Always” to all three survey questions. With a patient-centric focus, the primary team enhanced their communication skills with these patients using the instructions in the educational cards. The QI team conducted a second mock survey on the discharge day.

## Results

The project included 41 patients. The mean age was 54.7 years, ranging from 21.0 to 87.0 years. Thirty (73.2%) patients were male, while 11 (26.8%) participants were female. The mean length of stay was 5.6 days, with a median of four days (Table 2).

**Table 2:** Demographic variables

Total patients	41
Mean age (SD)	54.7 (16.78)
Median age	59
Sex	N (%)
Female	11 (26.8%)
Male	30 (73.2%)
Length of stay	Days
Mean (SD)	5.6 (3.72)
Median	4

**Figure 1:**
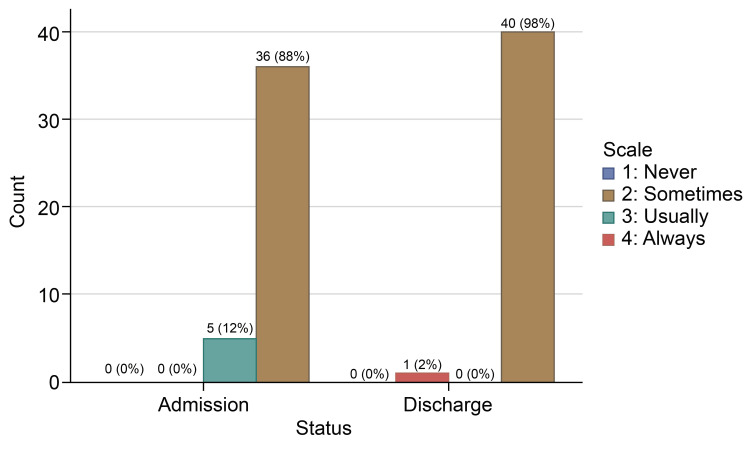
How often did the doctors treat you with respect?

**Figure 2:**
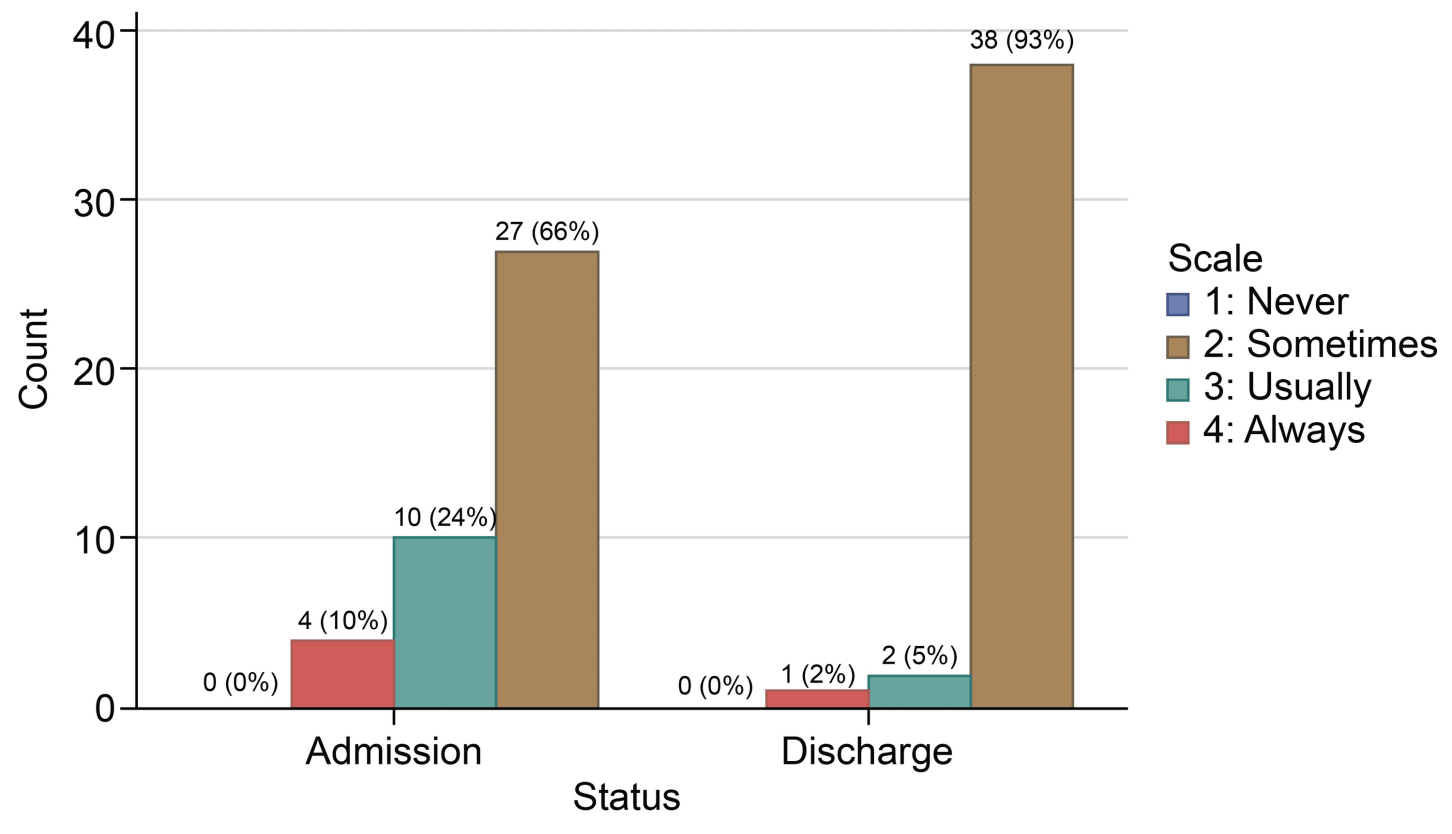
How often did the doctor listen to you?

**Figure 3:**
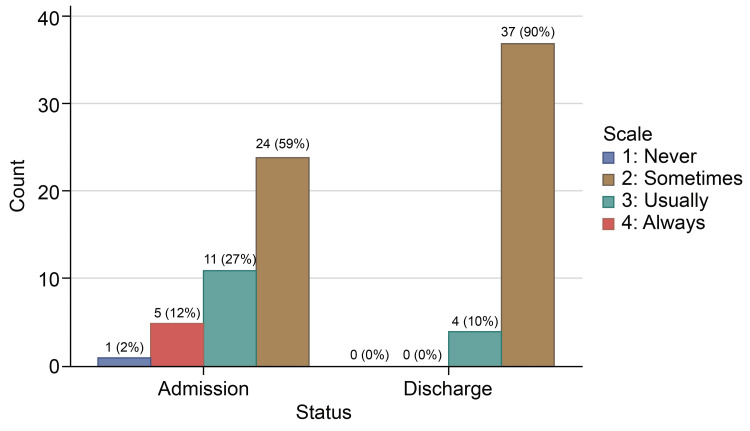
How often did doctors explain things in a language you can understand?

**Figure 4:**
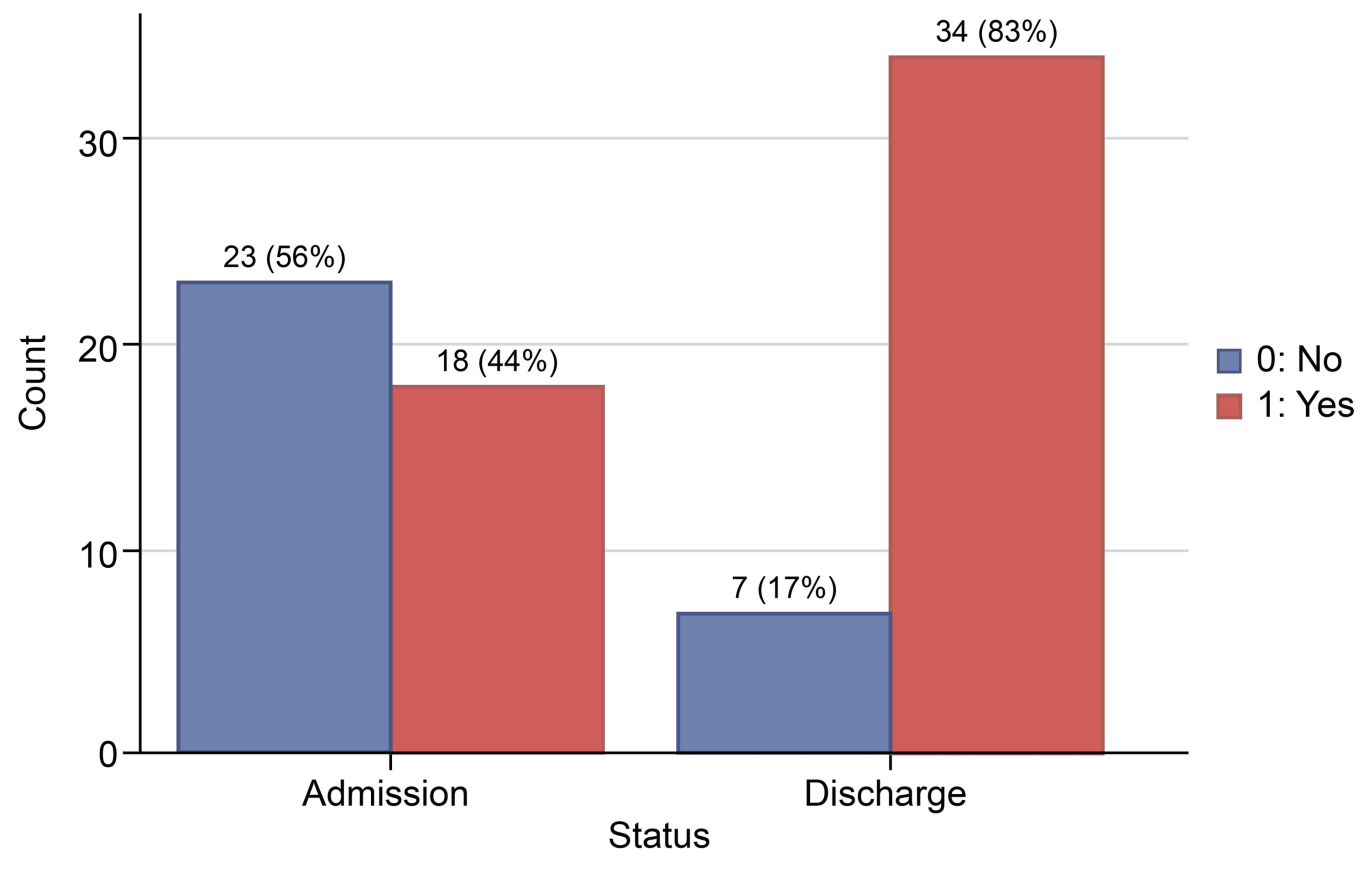
Patients who responded “always” to all three questions at admission and discharge time

In the responses for how often the doctor treated you with respect, 36 (87.8%) individuals reported “Always” feeling respected by their doctor on the admission survey, which increased to 40 (97.6%) patients on the discharge survey, while only one patient chose “Sometimes” on the discharge survey. None of the patients on admission or discharge survey opted for the “Never” choice for doctors’ respectful treatment (Table 3, Figure 1). Doctors' respectful treatment had the highest “Always” answers on the two surveys. About 27 (65.9%) individuals reported “Always” feeling listened to on the admission survey, which increased to 92.7% (38 patients) on the discharge survey. Two patients reported being “Usually” listened to, while one reported “Sometimes” (Table 4, Figure 2). In response to the question, “How often did the doctors explain things in a language you can understand?” 24 (58.5%) individuals reported “Always” understanding explanations on the admission survey, which improved to 37 (90.2%) individuals on the discharge survey (Table 5, Figure 3). One patient answered “Never” on the admission survey, but none picked his response on discharge. The number of patients who answered “Always” to all three questions increased from 18 (43.9%) patients on admission to 34 (82.9%) patients on discharge (Table 6, Figure 4).

**Table 3:** Doctor’s respectful treatment at admission and discharge time

Scale	Admission survey	Discharge survey
Never	0	0
Sometimes	0	1 (2.4%)
Usually	5 (12.2%)	0
Always	36(87.8%)	40 (97.6%)

**Table 4:** Doctor’s attentiveness at admission and discharge time

Scale	Admission survey	Discharge survey
Never	0	0
Sometimes	4 (9.8%)	1 (2.4%)
Usually	10 (24.4%)	2 (4.9%)
Always	27(65.9%)	38 (92.7%)

**Table 5:** Doctor’s explanatory performance at admission and discharge time

Scale	Admission survey	Discharge survey
Never	1 (2.4%)	0
Sometimes	5 (12.2%)	0
Usually	11 (26.8%)	4 (9.8%)
Always	24 (58.5%)	37 (90.2%)

**Table 6:** Patients who responded “always” to all three questions at admission and discharge time

	Admission survey	Discharge survey
No	23 (56.1%)	7 (17.1%)
Yes	18 (43.9%)	34 (82.9%)

## Discussion

Surveying patients early during the admission by asking the three HCAHPS doctor-patient communication questions and targeting patients with less than “always” answers increased patients’ satisfaction with their physicians’ communication by 39%. To our knowledge, double-surveying patients’ perceptions of their doctors’ communication early during admission and on discharge day have yet to be tested and published in English literature. This innovative approach allowed us to identify patients who are less satisfied with their physician communication early, alerting their physicians and refining communications with this group of patients by applying specific techniques taught in the QI educational sessions. Our intervention decreased the non “always” responses for all three questions. It increased the percentage of “always” answers except for one “sometimes” answer at discharge that was not chosen on the admission survey for doctors’ respectful treatment question. Few studies and QI projects were conducted in teaching services to improve the HCAPS score.

Banka et al. increased doctors’ patients’ HCAHPS communication scores by 6.6% through education conferences, providing feedback, and monthly recognition for internal medicine residents in the academic medical centers. The Connect with patients, Introduce yourself and your role, Communicate, Ask and anticipate, Respond, and Exit courteously (CICARE) framework was used in this study [9]. Simons et al. increased doctors’ communication scores by 9% by providing the patient’s face cards that listed the names and roles of attendees, residents, and interns [10]. Nabeel et al. saw a 6% improvement in doctors’ HCAHPS communication score by providing the internal medicine residents and attendings in a community hospital with the communication survey report completed by patients within 48 hours [11]. However, the most significant improvement of over 50% in the HCAPS doctor’s communication domain scores was reported in a QI by Tiperneni et al. [12]. Their intervention involved educating internal medicine residents on the Acknowledge, Introduce, Duration, Explanation, Thank you (AIDET) approach and conducting afternoon rounds with nursing staff to update, engage, and listen to the patients. Our intervention used a hybrid framework with items from CICARE and AIDET. Our project used an innovative approach that had never been tested and has the potential to improve patients’ satisfaction with doctors’ communication significantly. It can also be tested in other HCAHPS domains. The small number of patients in this QI is a limitation of this work. Patients’ experience with emergency room communication might affect the early survey score.

## Conclusions

Surveying patients about their physicians’ communication skills early during admission to identify patients with less than a perfect score and enhancing communication for this group of patients increased patients’ satisfaction scores with their physicians’ communication by 39%. This unique intervention has never been tested before, and it can potentially change how we conduct the HCAHPS survey. This novel approach should be tested in a large controlled study.
